# Open Ablation of the Left Ventricle During Implantation of a Left Ventricular Assist Device

**DOI:** 10.1016/j.atssr.2022.10.012

**Published:** 2022-10-29

**Authors:** Yuriy Hrytsyna, Jin-Hong Gerds-Li, Roland Heck, Pia Lanmüller, Leonard Pitts, Felix Hohendanner, Christoph Starck, Volkmar Falk, Evgenij V. Potapov

**Affiliations:** 1 Department of Cardiothoracic and Vascular Surgery, German Heart Center Berlin, Berlin, Germany; 2 Department of Internal Medicine and Cardiology, German Heart Institute, Berlin, Germany; 3 DZHK (German Centre for Cardiovascular Research), Partner Site Berlin, Berlin, Germany; 4 Berlin Institute of Health (BIH), Berlin, Germany; 5 Department of Cardiovascular Surgery, Charité-Universitaetsmedizin Berlin, Berlin, Germany; 6 Department of Health Sciences and Technology, Translational Cardiovascular Technologies, Institute of Translational Medicine, Swiss Federal Institute of Technology (ETH) Zurich, Zurich, Switzerland

## Abstract

This report describes the successful treatment of persistent ventricular tachycardia in a 66-year-old patient with ischemic cardiomyopathy during left ventricular assist device (LVAD) implantation. Recurrent episodes of ventricular tachycardia led to cardiac decompensation necessitating LVAD implantation. After opening the left ventricle, an AtriCure Isolator Synergy OLL2 radiofrequency clamp was used to perform 4 sets of transmural lesions. Transmurality was observed in all lesions. A HeartMate 3 (Abbott) LVAD was implanted in standard fashion. The lesions were connected with an epicardial ablation line using a radiofrequency catheter. The patient was discharged home without complications and with a stable rhythm.

Continuous-flow left ventricular assist device (LVAD) implantation is an established treatment option for end-stage heart failure. Ventricular tachyarrhythmias (VTs) are estimated to occur in 60% of patients undergoing LVAD implantation. VTs are also known complications after LVAD implantation, occurring in >35% of patients despite unloading of the left ventricle (LV),^1^ with 10% of patients developing electrical storm associated with high mortality.^2^ VTs are mostly caused by the arrhythmogenic substrate that develops as heart failure progresses.^3^ Pre-LVAD VTs were found to be the most important predictors of post-LVAD ventricular arrhythmias.^4^ In this report, we present a new option for the treatment of refractory VT during implantation of a durable LVAD using an AtriCure Isolator Synergy OLL2 radiofrequency (RF) clamp.

A 66-year-old man was admitted with a relapse of slow VT and electrical storm. He developed ischemic cardiomyopathy after a posterior wall myocardial infarction in 2011 with subsequent formation of an apical ventricular aneurysm with low left ventricular ejection fraction. Because of several episodes of VT, an implantable cardioverter-defibrillator was placed in 2011 and upgraded to a cardiac resynchronization therapy defibrillator in 2020. Since 2011, several LV ablations were performed because of a relapse of VT. During the current hospitalization, 3-dimensional mapping of the LV showed a macroreentrant circuit in the area of the inferobasal scar. Three-dimensional RF ablation of the LV was performed, and a ventricular tachycardia isthmus was transected from the scar area to the mitral valve. Despite initial success, on the following day the patient experienced a VT relapse as well as signs of cardiogenic shock with a need for inotropic support.

Impella 5.0 (Abiomed) was implanted. Echocardiography showed global hypokinesia and a left ventricular ejection fraction of 20%. After 4 days on Impella, implantation of a durable LVAD with concomitant epicardial ablation of the LV was considered. The patient gave his consent for off-label use of the clamp for myocardial ablation with the goal of treating the recurrent VT. After median sternotomy and initiation of cardiopulmonary bypass, the Impella 5.0 device was explanted. Epicardial ablation employing an RF catheter was performed over the posterior wall of the LV from the mitral valve annulus over the scar toward the LV apex. After this procedure, the LV apex was cored using a HeartMate 3 coring tool (Abbott). The Isolator Synergy OLL2 clamp (AtriCure) was inserted into the left ventriculotomy, and 4 sets of transmural lesions were performed (Figures 1 and 2): No. 1 was placed parallel to the left anterior descending artery toward the proximal aspect; No. 2, over the lateral wall; No. 3, over the posterior wall; and No. 4, parallel to the posterior descending artery.

**Figure 1:**
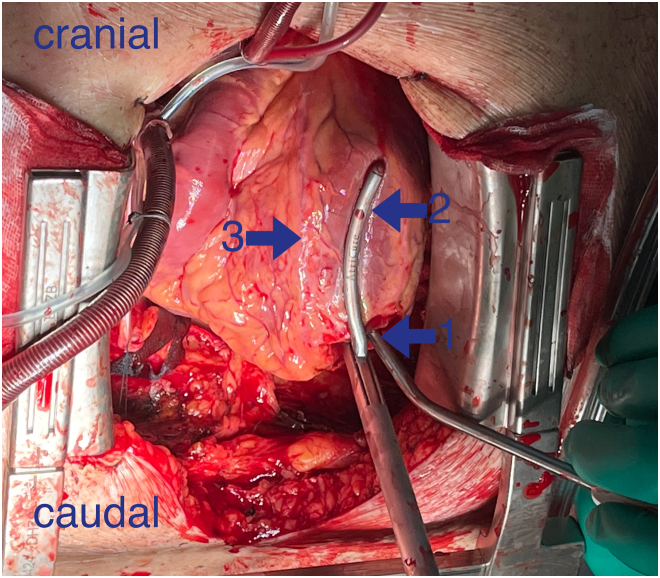
Ablation of the anterior left ventricle wall. (1) Left ventriculotomy in the apex region. (2) OLL2 radiofrequency clamp. (3) Left anterior descending artery.

**Figure 2:**
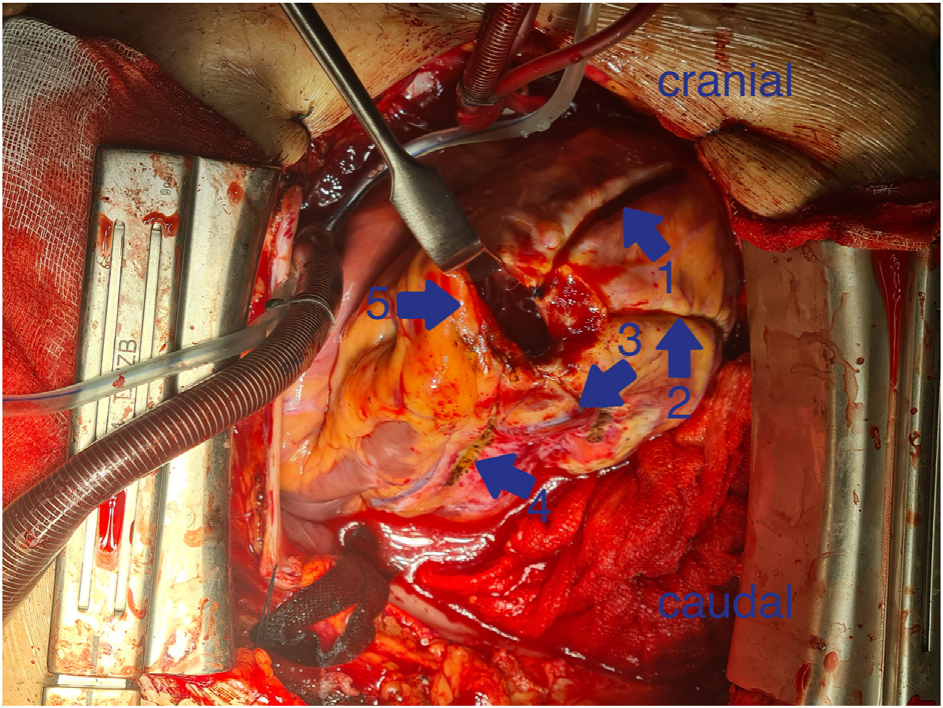
Complete set of lesions after left ventricle ablation with the OLL2 radiofrequency clamp. (1) Lesion parallel to the left anterior descending artery toward the proximal aspect. (2) Lateral wall lesion. (3) Posterior wall lesion. (4) Lesion parallel to the posterior descending artery. (5) Left ventriculotomy in the apex region.

All lesions were controlled to rule out LV wall transection and to check for signs of transmurality. The fixation ring was then sewn over the LV opening using 12 polypropylene (3-0 Prolene) U-shaped sutures reinforced with pledgets as described previously.^5^ After that, lesion lines were connected using the RF TC catheter (Biosense Webster) parallel to the fixation ring from the left anterior descending artery to the posterior descending artery (Figure 3). The HeartMate 3 LVAD was then implanted in standard fashion^5^ and started; cardiopulmonary bypass was terminated in a stable sinus rhythm with good RV function.

**Figure 3:**
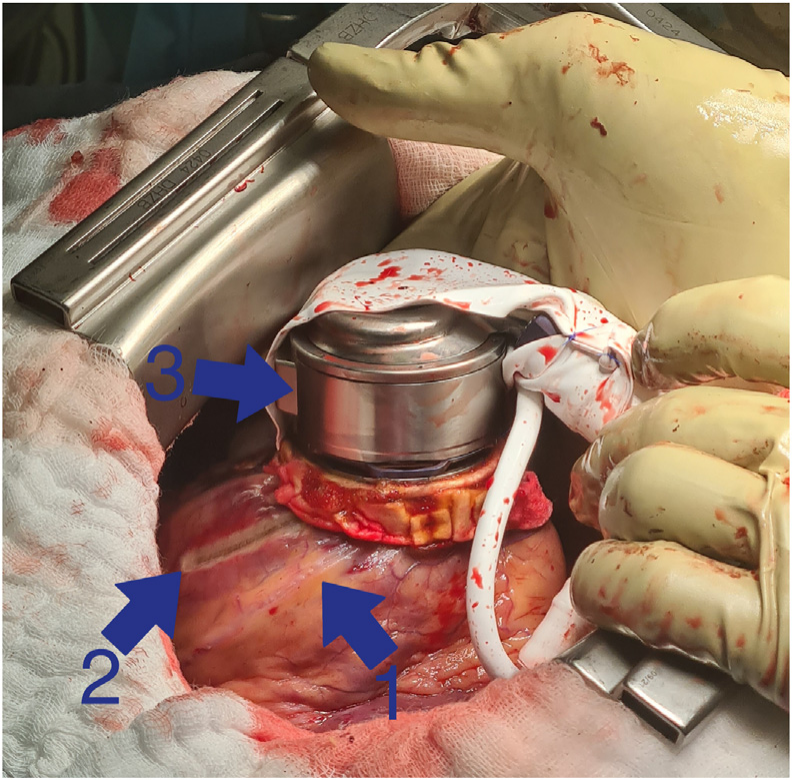
Final result after left ventricular assist device implantation. (1) Left anterior descending artery. (2) Lesion parallel to the left anterior descending artery toward the proximal aspect. (3) HeartMate 3 left ventricular assist device attached to a fixation ring.

The patient was successfully discharged home and presented with a stable rhythm after 148 days of follow-up. No complications were observed in the postoperative period.

The case is presented in the form of a Supplemental Video.

## Comment

We report the use of an Isolator Synergy OLL2 clamp for the surgical treatment of recurrent VT in a patient undergoing LVAD implantation. The procedure resulted in a stable rhythm without complications during the follow-up.

VT is a serious condition in patients undergoing LVAD implantation that, if left untreated, leads to impairment of right ventricular function, resulting in cardiac decompensation despite ongoing LVAD support. The Isolator Synergy OLL2 clamp was approved for use in the surgical treatment of atrial fibrillation.^6^ The system consists of a clamp with 2 jaws, each containing 2 RF electrodes, as well as a sensing and ablating unit. The energy is delivered between the jaws in an alternating fashion with a frequency of 267 Hz and a maximum of 28 W of power.

Our case report showed that placement of transmural lesions on LV myocardial tissue is safe. The ablation lines remained stable after LVAD implantation. The question whether ablation of all myocardial layers in a tissue that is already remodeled by ischemic or dilated cardiomyopathy is associated with a greater risk of perforation remains to be answered in an experimental setting. Histologic postmortem tissue analysis in an animal model after ablation would show the depth of the ablation, prove the transmurality, and identify signs of risk of perforation. Another risk during the ablation of the LV is the possibility of causing damage to the coronary arteries. This can be avoided by placing ablation lines between coronary arteries at a sufficient distance.

The recurrence rate after conventional interventional ablation in LVAD patients is estimated to range between 15% and 86%.^4^ The question as to what set of lesions leads to freedom from VT recurrence as well as concerning the value of additionally connecting lesions remains unanswered.

In conclusion, the described surgical ablation led to the formation of a transmural scar around the apex and may contribute to a longer or lasting recurrence-free state after LVAD implantation.

The Video can be viewed in the online version of this article [https://doi.org/10.1016/j.atssr.2022.10.012] on http://www.annalsthoracicsurgery.org.
